# The role of heavy metals in the development of colorectal cancer

**DOI:** 10.1186/s12885-023-11120-w

**Published:** 2023-07-03

**Authors:** Yongsheng Li, Jingwei Lou, Shaozhong Hong, Dengfeng Hou, Yandong Lv, Zhiqiang Guo, Kai Wang, Yue Xu, Yufeng Zhai, Hongzhou Liu

**Affiliations:** 1 Department of Colorectal Surgery, Heping Hospital Affiliated to Changzhi Medical College, Changzhi, 046000 China; 2 Shanghai Biotecan Pharmaceuticals Co., Ltd, Shanghai, 201204 China

**Keywords:** Colorectal cancer, Microsatellite instability, Heavy metals, Trace elements, *BRAF V600E*, Genetic polymorphism, Tumor markers

## Abstract

**Objective:**

To investigate the relationship among 18 heavy metals, microsatellite instability (MSI) status, *ERCC1*, *XRCC1 (rs25487)*, *BRAF V600E* and 5 tumor markers and their role in the development of colorectal cancer (CRC).

**Methods:**

A total of 101 CRC patients and 60 healthy controls were recruited in the present study. The levels of 18 heavy metals were measured by ICP-MS. MSI status and the genetic polymorphism were determined by PCR (FP205-02, Tiangen Biochemical Technology Co., Ltd., Beijing, China) and Sanger sequencing. Spearman’s rank correlation was used to analyze the relationship among various factors.

**Results:**

The level of selenium (Se) was lower in the CRC group compared with the control group (p < 0.01), while vanadium (V), arsenic (As), tin (Sn), barium (Ba) and lead (Pb) were higher (p < 0.05), chromium (Cr) and copper (Cu) were significantly higher (p < 0.0001) in the CRC group than those in the control group. Multivariate logistic regression analysis indicated that Cr, Cu, As and Ba were the risk factors for CRC. In addition, CRC was positively correlated with V, Cr, Cu, As, Sn, Ba and Pb, but negatively correlated with Se. MSI was positively correlated with *BRAF V600E*, but negatively correlated with *ERCC1*. *BRAF V600E* was positively correlated with antimony (Sb), thallium (Tl), CA19-9, NSE, AFP and CK19. *XRCC1 (rs25487)* was found to be positively correlated with Se but negatively correlated with Co. The levels of Sb and Tl were significantly higher in the *BRAF V600E* positive group compared to the negative group. The mRNA expression level of *ERCC1* was significantly higher (P = 0.035) in MSS compared to MSI. And there was a significant correlation between *XRCC1 (rs25487)* polymorphism and MSI status (P<0.05).

**Conclusion:**

The results showed that low level of Se and high levels of V, As, Sn, Ba, Pb, Cr, and Cu increased the risk of CRC. Sb and Tl may cause *BRAF V600E* mutations, leading to MSI. *XRCC1 (rs25487)* was positively correlated with Se but negatively correlated with Co. The expression of *ERCC1* may be related to MSS, while the *XRCC1 (rs25487)* polymorphism is related to MSI.

## Introduction

Colorectal cancer (CRC) is the third most common malignant tumor of the digestive tract worldwide, and it is also the main cause of cancer-related deaths, posing a significant threat to human health [1, 2]. Researchers have found that some molecular markers, such as microsatellite instability (MSI) and B-type Raf kinase (BRAF) mutations, display associations with survival and are used as important prognostic factors for intrinsic CRC subtypes [3, 4].

MSI is caused by functional defects such as deletion or alteration of DNA mismatch repair (MMR) protein, and is considered as a prognostic marker for CRC [5, 6]. MSI occurs in about 15% of CRC patients, and generally associated with a better clinical outcome of CRC compared to microsatellite stable (MSS) [4].

BRAF is a member of the RAF family and an important component in the mitogen activated protein kinase (MAPK) pathway, usually activated by its mutations [4]. BRAF mutations are major carcinogenic factors in CRC [7]. 80% of BRAF mutations are missense mutations that occur in codon 600, and missense mutations are transitions from valine (V) to glutamic acid (E) at codon 600 caused by c. 1799T > A transposition (V600E) [8]. *BRAF V600E* mutations occur in 5–10% of CRC patients and are associated with poor prognosis [9, 10]. Studies have shown that the relative mortality rate of *BRAF V600E* mutations has increased nearly 2-fold higher than that of wild-type BRAF [10].

Genetic polymorphisms in DNA repair genes can impede DNA repair ability, potentially leading to the development of cancers such as CRC [11]. Among the identified polymorphisms of DNA repair genes, excision repair cross-complementing group 1 (*ERCC1*) and X-ray repair cross complementing group 1 (*XRCC1*) play an indispensable role in nucleotide excision repair and may be related to the incidence rate of some cancers [12, 13]. As a highly conserved enzyme, *ERCC1* participates in the key steps of nucleotide excision repair, and its expression level is a major predictor of cancer response to platinum‑based chemotherapy [14, 15]. *XRCC1* is associated with base-excision repair and single strand break repair [16]. As a common genetic polymorphism in the *XRCC1* gene, substitution of *XRCC1* Arg to Gln at codon 399 (rs25487) can contribute to impaired DNA repair activity by altering the function of the *XRCC1* protein [17]. And results of Hosseini et al. showed that the polymorphism of *XRCC1 (rs25487)* may be associated with an increased risk of CRC [13].

Despite significant progress in treatment over the past few years, the prognosis of patients with metastatic CRC remains poor, with a 5-year overall survival rate (OS) of less than 15% [18]. With the increasing incidence rate of CRC, the World Health Organization recommends focusing on early detection and follow-up after surgery to prolong the survival of patients [19]. In recent years, serum tumor markers can not only be used for early screening and diagnosis of cancer, but also play an important role in evaluating treatment response, predicting recurrence, and assessing prognosis and survival [20]. The commonly used tumor markers for the diagnosis and evaluation of CRC patients include carcinoembryonic antigen (CEA), neuron-specific enolase (NSE), cancer antigen (CA)19 − 9 and alpha-fetoprotein (AFP) [21–24]. In addition, as an epithelial cytoskeleton marker, cytokeratin 19 (CK19) may serve as a prognostic indicator for cancer patients, but there are few relative studies in the context of CRC [25].

In addition, it is well known that cancer is a complex process influenced by multiple factors [26]. It is reported that 80% of cancer cases are caused by environmental factors, such as trace elements and heavy metals [27]. Although trace elements can enhance immunity, their deficiency or excess can lead to metabolic and cell growth disorders and tumorigenesis [27]. As early as 1975, Schwartz reviewed the role of trace elements including selenium (Se), zinc (Zn), and copper (Cu) in cancer, and discussed their potential utility as diagnostic or prognostic markers [28]. In addition, Nawi et al. reported that the concentrations of manganese (Mn), cadmium (Cd), Cu, magnesium (Mg), lead (Pb), chromium (Cr) and Zn in metastatic patients were increased compared to the general population [29]. Sohrabi et al. have also demonstrated that the levels of heavy metals and trace elements including thallium (Tl), Zn, Pb, Cr and Cu in CRC cancer tissues were significantly higher than those in healthy ones [30]. However, the mechanisms underlying role of heavy metals in the progression of CRC and the interactions among these heavy metals are not fully understood. Therefore, the purpose of this study was to elucidate the association among 18 heavy metals, MSI status, 5 tumor markers, and genetic polymorphisms and their role in the development of CRC.

## Materials and methods

### Study characteristics

A total of 101 CRC patients and 60 healthy controls were recruited at Heping Hospital Affiliated to Changzhi Medical College, from January 2020 to February 2021. The study was approved by the Ethics Committees of Heping Hospital Affiliated to Changzhi Medical College (Approval number: 2,018,006), and was conducted in accordance with the standards of Declaration of Helsinki. Prior to participation, informed consent was obtained from all subjects and/or their legal guardian(s).

Inclusion criteria: Clinically diagnosed patients with primary colorectal cancer.

Exclusion criteria: (1) Combined with other major diseases; (2) Incomplete clinical data or missing visits.

### DNA extraction

Genomic DNA was extracted using the QIAamp DNA FFPE Tissue Kit (56,404, QIAGEN, Germany) according to the manufacturer’s instructions. Nano Drop2000 UV-Vis Spectrophotometer (Thermo, USA) was used to measure the purity and concentration of DNA.

### MSI, SNP genotyping and mutation analysis

MSI status was determined by PCR (FP205-02, Tiangen Biochemical Technology Co., Ltd., Beijing, China) using a panel of five microsatellite markers including three dinucleotide (D2S123, D5S346, D17S250) and two mononucleotide (BAT25, BAT26) repeats, and the PCR products after amplification were detected and analyzed by capillary electrophoresis with ABI 3730XL DNA Analyzer (ABI, USA) [31]. Microsatellite instability-high (MSI-H) was defined when there were two or more instability markers, MSI-L was defined when there was only one instability marker, and if there was no instability among the five markers, it was judged to be MSS.

The SNP of *XRCC1 (rs25487)* was determined by PCR and Sanger sequencing by ABI 3730XL DNA Analyzer (ABI, USA).

The human NRAS mutation detection kit (YZYMT-019-C, Wuhan YZY Medical Science & Technology Co., Ltd., Wuhan, China) and the human *BRAF V600E* detection kit (SMD-02-026, Beijing SinoMDgene Technology Co., Ltd., Beijing, China) were used to detect the relevant mutation sites.

### Quantitative real-time PCR (qPCR)

The mRNA expression level of *ERCC1* was measured by qPCR using ABI‑7500 real‑time PCR system (Thermo Fisher Scientific, Inc.). Thermocycling conditions were set as follows: Pre‑denaturation at 95˚C for 15 min; followed by 45 cycles of amplification for 15 s at 95 °C and 30 s at 60 °C; GAPDH was used as an internal standard for *ERCC1* mRNA expression. The primer sequences were as follows: *ERCC1* forward, 5´-GGGAATTTGGCGACGTAATTC-3´, and reverse, 5´-GCGGAGGCTGAGGAACAG-3´; GAPDH forward, 5´-GCCACATCGCTCAGACACC-3´, and reverse, 5´-GATGGCAACAATATCCACTTTACC-3´. The mRNA expression of *ERCC1* was obtained by the comparative ΔCt method.

### ICP-MS experiment

Approximately 2ml of whole blood was collected from each participant, centrifuged at 3000 rpm for 10 min to separate the serum, and then stored at -20˚C until further analysis. According to the manufacturer’s instructions, the levels of 18 heavy metals including arsenic (As), barium (Ba), cadmium (Cd), cobalt (Co), chromium (Cr), copper (Cu), gallium (Ga), mercury (Hg), manganese (Mn), nickel (Ni), lead (Pb), antimony (Sb), selenium (Se), tin (Sn), strontium (Sr), thallium (Tl), vanadium (V) and zinc (Zn) were determined by ICP-MS (Agilent 7800) [32].

### Statistical analysis

Statistical analysis was performed using SPSS 22.0. Chi-square test was used to evaluate the distribution difference of categorical variables between groups and was expressed in numbers (percentages). Normal distribution of continuous variables was preliminarily analyzed by Kolmogorov-Smirnov test, and the differences among groups were compared by Kruskal - Wallis H test or Mann - Whitney U as appropriate, and described as mean ± standard deviation (SD). Risk factors of CRC were analyzed by binary and multiple logistic regression analysis. And the relationship among various factors were analyzed with R studio and Spearman’s rank correlation. R studio, GraphPad Prism 6.0 and Adobe Illustrator 2020 were used to generate the graph. *P*-value < 0.05 (two- tailed) was deemed to be statistically significant.

## Results

### Clinical characteristics of the study population

**Fig. 1:**
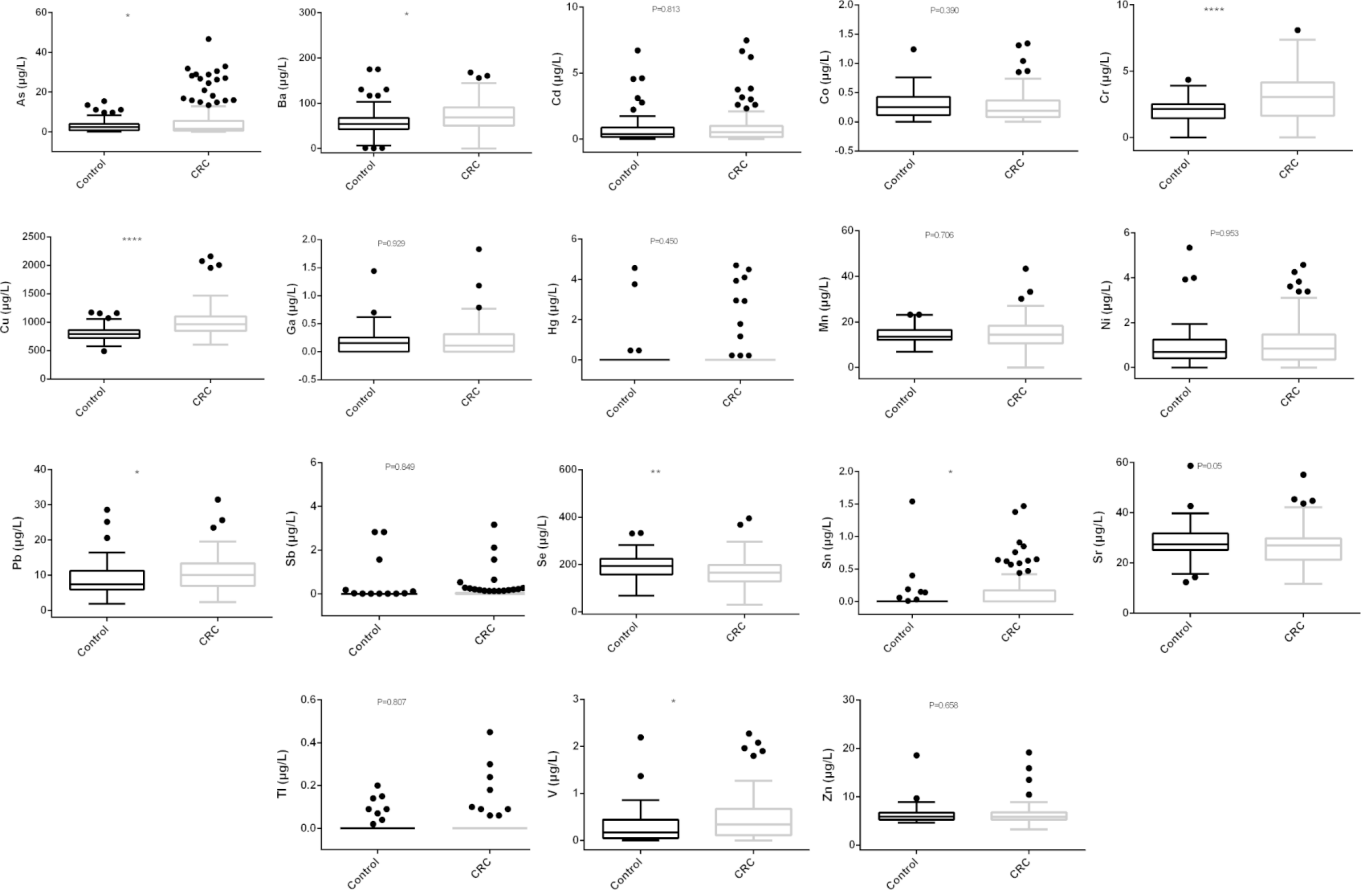
Comparison of 18 heavy metals levels between the control group and CRC group. *, P < 0.05; **, P < 0.01; ***, P < 0.001; ****, P < 0.0001

A total of 161 participants, consisting of 101 CRC patients and 60 healthy controls were enrolled in this study, the clinical characteristics were shown in Table 1. There was no significant difference in the concentration of heavy metals (including Mn, Co, Ni, Zn, Ga, Sr, Cd, Sb, Hg and Tl) between CRC group and healthy group (p > 0.05). The level of Se was lower in the CRC group compared with the control group (p < 0.01), while V, As, Sn, Ba and Pb were higher (p < 0.05), Cr and Cu were significantly higher (p < 0.0001) in the CRC group than those in the control group (Table 1; Fig. 1). The results showed that low level of Se and high levels of V, As, Sn, Ba, Pb, Cr and Cu increased the risk of CRC.

**Table 1:** Clinical characteristics of the CRC patients and controls

Variables	Controls	CRC	*p* value
Age (years)	45.13 ± 16.76	60.77 ± 10.28	<0.001
Male (n, %)	16 (26.67)	54 (53.47)	0.001
BMI (kg/m^2^)	24.99 ± 4.64	23.87 ± 3.54	0.087
Smoking (n, %)	7 (11.67)	37 (36.63)	0.001
Drinking (n, %)	21 (35)	12 (11.88)	<0.001
Diabetes (n, %)	2 (3.33)	6 (5.94)	0.462
Hypertension (n, %)	13 (21.67)	32 (31.68)	0.171
V (ug/L)	0.29 ± 0.37	0.49 ± 0.63	0.024
Cr (ug/L)	1.94 ± 0.80	3.09 ± 1.75	<0.001
Mn (ug/L)	14.52 ± 3.91	14.88 ± 6.41	0.695
Co (ug/L)	0.29 ± 0.24	0.26 ± 0.26	0.390
Ni (ug/L)	1.16 ± 1.55	1.14 ± 1.18	0.953
Cu (ug/L)	815.56 ± 137.92	1003.77 ± 272.29	<0.001
Zn (ug/L)	6.41 ± 2.01	6.26 ± 2.12	0.658
Ga (ug/L)	0.19 ± 0.24	0.19 ± 0.27	0.929
As (ug/L)	3.43 ± 3.52	6.22 ± 9.88	0.037
Se (ug/L)	194.63 ± 53.60	167.33 ± 57.89	0.003
Sr (ug/L)	29.57 ± 11.57	26.57 ± 7.66	0.050
Cd (ug/L)	0.95 ± 1.81	0.89 ± 1.32	0.813
Sn (ug/L)	0.04 ± 0.21	0.18 ± 0.48	0.042
Sb (ug/L)	0.13 ± 0.55	0.11 ± 0.41	0.849
Ba (ug/L)	60.09 ± 35.56	74.68 ± 33.60	0.010
Hg (ug/L)	0.48 ± 2.64	0.26 ± 0.95	0.450
Tl (ug/L)	0.01 ± 0.04	0.02 ± 0.06	0.806
Pb (ug/L)	9.03 ± 5.24	10.90 ± 5.91	0.044

### Logistic regression analysis of risk factors in CRC development

Univariate logistic regression analysis was used to identify the risk factors of CRC development to evaluate independent indicators related to CRC. In the unadjusted model, age (OR = 1.087, P < 0.001), gender (OR = 3.160, P = 0.001) and smoking (OR = 4.377, P < 0.001) were identified as the risk factors for CRC among clinical factors. For the heavy metals, V (OR = 2.747, P = 0.028), Cr (OR = 1.869, P < 0.001), Cu (OR = 1.006, P < 0.001), As (OR = 1.053, P = 0.046), Sn (OR = 9.188, P = 0.042), Ba (OR = 1.013, P = 0.012), and Pb (OR = 1.069, P = 0.049) were identified as the risk factors, while Se (OR = 0.991, P = 0.005) was considered as the protective factor (Table 2). Moreover, after adjusting the clinical influencing factors (including age, gender, smoking, drinking), the multivariate logistic regression analysis was conducted to further investigate the independent correlation between heavy metals and the development of CRC. Results showed that age (OR = 1.071, P < 0.001), smoking (OR = 14.517, P = 0.018), Cr (OR = 2.523, P < 0.001), Cu (OR = 1.005, P = 0.001), As (OR = 1.074, P = 0.039), and Ba (OR = 1.021, P = 0.003) were still the risk factors for CRC (Table 3).

**Table 2:** Risk factors of CRC by logistic regression analysis. CI, confidence interval; OR, odds ratio

Variables	β	SE	Wald	*p* value	OR	95% CI
Age	0.084	0.015	32.807	<0.001	1.087	1.057–1.119
Gender	1.150	0.354	10.586	0.001	3.160	1.580–6.318
BMI	-0.070	0.041	2.843	0.092	0.933	0.860–1.011
Smoking	1.476	0.452	10.666	0.001	4.377	1.805–10.617
Drinking	-1.385	0.410	11.425	0.001	0.250	0.112–0.559
Diabetes	0.605	0.833	0.527	0.468	1.832	0.358–9.379
Hypertension	0.517	0.379	1.856	0.173	1.677	0.797–3.527
V	1.011	0.460	4.829	0.028	2.747	1.115–6.767
Cr	0.625	0.149	17.618	<0.001	1.869	1.396–2.502
Mn	0.012	0.030	0.155	0.694	1.012	0.955–1.072
Co	-0.550	0.639	0.739	0.390	0.577	0.165–2.021
Ni	-0.007	0.123	0.004	0.952	0.993	0.780–1.263
Cu	0.006	0.001	22.037	<0.001	1.006	1.004–1.009
Zn	-0.034	0.077	0.197	0.657	0.966	0.830–1.124
Ga	0.057	0.632	0.008	0.928	1.059	0.307–3.653
As	0.052	0.026	3.995	0.046	1.053	1.001–1.108
Se	-0.009	0.003	7.856	0.005	0.991	0.986–0.997
Sr	-0.034	0.018	3.518	0.061	0.966	0.932–1.002
Cd	-0.025	0.106	0.057	0.812	0.975	0.792–1.201
Sn	2.218	1.091	4.135	0.042	9.188	1.084–77.902
Sb	-0.066	0.346	0.037	0.848	0.936	0.475–1.844
Ba	0.013	0.005	6.256	0.012	1.013	1.003–1.024
Hg	-0.068	0.095	0.520	0.471	0.934	0.776–1.125
Tl	0.755	3.065	0.061	0.805	2.127	0.005-864.245
Pb	0.066	0.034	3.881	0.049	1.069	1.000-1.142

**Table 3:** Multivariate logistic regression analysis of the independent correlation between 18 heavy metals and CRC risk, by adjusting clinical risk factors. CI, confidence interval; OR, odds ratio

Variables	β	SE	Wald	*p* value	OR	95% CI
Age	0.069	0.016	19.505	<0.001	1.071	1.039–1.105
Gender	0.769	0.609	1.595	0.207	2.158	0.654–7.116
Smoking	2.675	1.132	5.590	0.018	14.517	1.580-133.369
Drinking	-3.289	1.070	9.451	0.002	0.037	0.005–0.304
V	0.574	0.491	1.368	0.242	1.775	0.678–4.644
Cr	0.925	0.235	15.513	<0.001	2.523	1.592–3.999
Cu	0.005	0.001	10.387	0.001	1.005	1.002–1.007
As	0.072	0.035	4.269	0.039	1.074	1.004–1.150
Se	-0.005	0.004	1.557	0.212	0.995	0.987–1.003
Sn	1.598	1.047	2.328	0.127	4.942	0.635–38.489
Ba	0.021	0.007	8.957	0.003	1.021	1.007–1.035
Pb	-0.052	0.043	1.471	0.225	0.949	0.872–1.033

Model: adjusted for age, gender, smoking, drinking

### Correlation analysis among CRC, clinical variables and 18 heavy metals

**Fig. 2:**
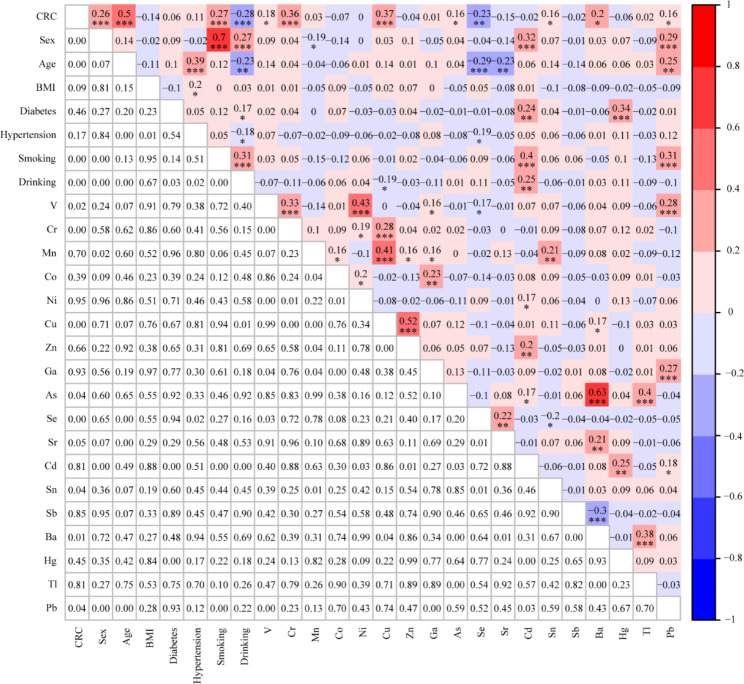
Spearman correlation among CRC, clinical variables and 18 heavy metals. *P < 0.05, **P < 0.01, ***P < 0.001

Spearman correlation analysis among CRC, clinical variables and 18 heavy metals were illustrated in Fig. 2. CRC was positively correlated with age (r = 0.5, p < 0.001), V (r = 0.18, p < 0.05), Cr (r = 0.36, p < 0.001), Cu (r = 0.37, p < 0.001), As (r = 0.16, p < 0.05), Sn (r = 0.16, p < 0.05), Ba (r = 0.2, p < 0.05), and Pb (r = 0.16, p < 0.05), but negatively correlated with Se (r = − 0.23, P < 0.01). Moreover, the correlation between these heavy metals showed that V was positively correlated with Cr (r = 0.33, P < 0.001), Ni (r = 0.43, P < 0.001), Pb (r = 0.28, P < 0.001), and Ga (r = 0.16, P < 0.05), but negatively correlated with Se (r = − 0.17, p < 0.05); Cr was positively correlated with Cu (r = 0.28, P < 0.001) and Ni (r = 0.19, P < 0.05); As was positively correlated with Ba (r = 0.63, P < 0.001), Tl (r = 0.4, P < 0.001), and Cd (r = 0.17, P < 0.05); Cd was positively correlated with Hg (r = 0.25, P < 0.01) and Pb (r = 0.18, P < 0.05). In addition to the above, other heavy metals also showed significant correlations, as shown in Fig. 2.

### Characteristics of CRC patients

According to the MSI test results, there were 30 MSI and 71 MSS in 101 patients with CRC, and the characteristics were shown in Table 4. There was no significant difference in age, sex, BMI, smoking, drinking, history of diabetes, history of hypertension, history of CAD, degree of tumor differentiation, family history of tumor, nerve invasion and distant metastasis between MSI and MSS. However, tumor stage (P = 0.016), tumor size (P = 0.028), vascular invasion (P = 0.035), lymph node metastasis (P = 0.016), *BRAF V600E* (P = 0.035) and *NRAS* Codon 12/13 (P = 0.021) were significantly different between the two groups. And the mRNA expression level of *ERCC1* was significantly higher (P = 0.035) in MSS compared to MSI.

**Table 4:** Clinical characteristics of 101 CRC patients in this study

Variables	MSI	MSS	*p* value
Age (years)	61.07 ± 9.97	60.65 ± 10.48	0.853
Sex			
Male	13	37	0.420
Female	17	34	
BMI (kg/m^2^)	23.50 ± 3.04	24.03 ± 3.74	0.500
Smoking			0.648
Yes	12	25	
No	18	46	
Drinking			0.334
Yes	5	7	
No	25	64	
Diabetes			0.471
Yes	1	5	
No	29	66	
Hypertension			0.243
Yes	12	20	
No	18	51	
CAD			0.946
Yes	2	5	
No	28	66	
Tumor stage			0.016
I-II	21	31	
III-IV	9	40	
Degree of tumor differentiation			0.817
Low, middle-low	8	19	
Middle, high	21	51	
NA	1	1	
Family history of tumor			0.260
Yes	2	3	
No	27	68	
NA	1	0	
Tumor size (cm)			0.028
≤ 3	2	0	
>3	28	71	
Nerve invasion			0.076
Yes	2	15	
No	28	56	
Vascular invasion			0.035
Yes	2	18	
No	27	53	
NA	1	0	
Lymph node metastasis			0.016
Yes	9	40	
No	21	31	
Distant metastasis			0.292
Yes	2	10	
No	28	61	
*BRAF V600E*			0.035
Mutation	4	1	
Wild	26	69	
NA	0	1	
*NRAS* Codon 12/13			0.021
Mutation	3	0	
Wild	27	70	
NA	0	1	
mRNA expression level of *ERCC1*	1.71 ± 2.30	2.75 ± 2.20	0.035

### Correlation analysis among MSI, BRAF V600E, ERCC1, XRCC1 (rs25487), 5 biomarkers, and 18 heavy metals

**Fig. 3:**
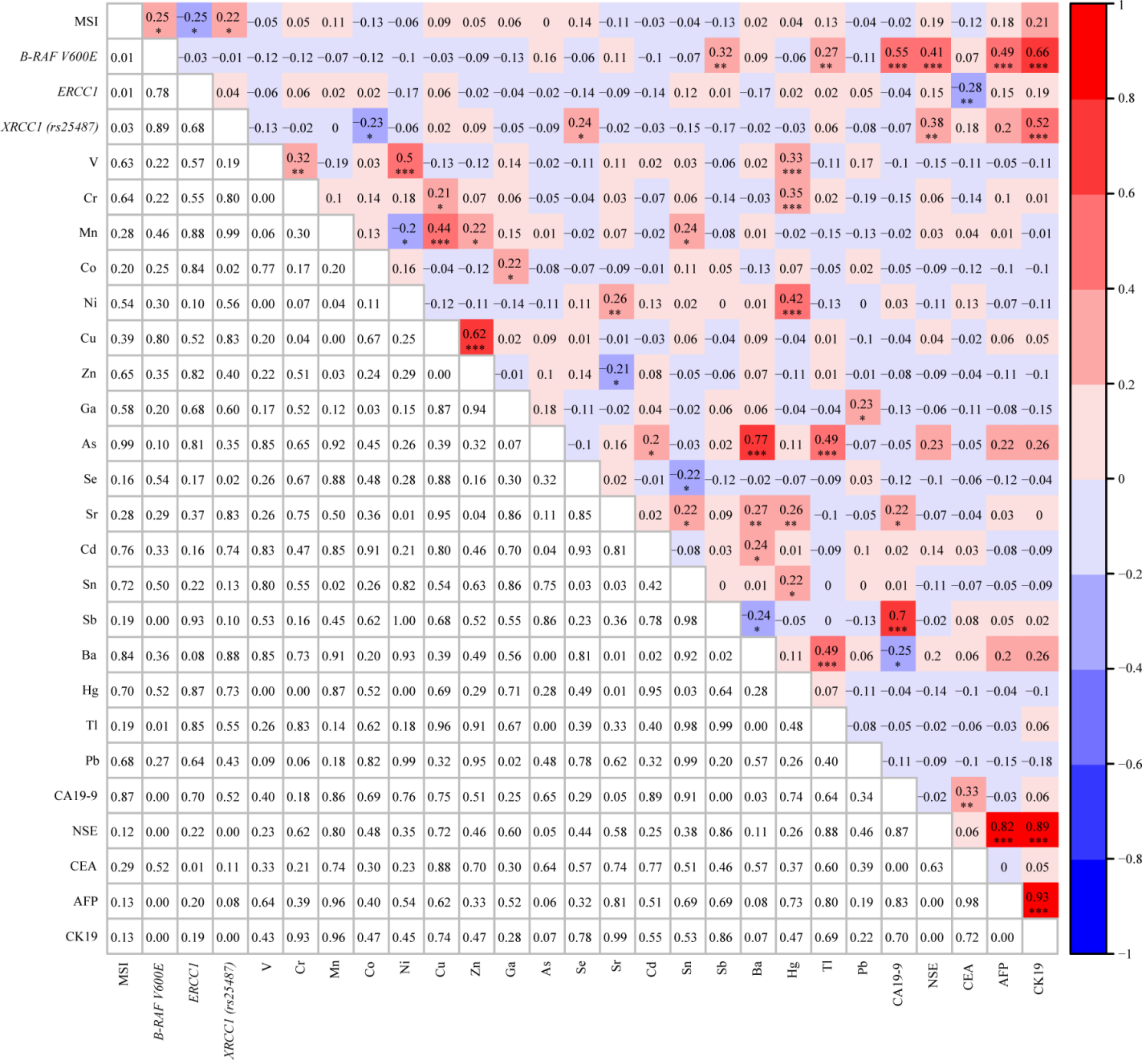
Spearman correlation among MSI, *BRAF V600E, ERCC1, XRCC1 (rs25487)*, 5 biomarkers and 18 heavy metals. *P < 0.05, **P < 0.01, ***P < 0.001

Spearman correlation analysis among MSI, *BRAF V600E*, *ERCC1*, 5 biomarkers, and 18 heavy metals were illustrated in Fig. 3. MSI was positively correlated with *BRAF V600E* (r = 0.25, p < 0.05) and *XRCC1 (rs25487)* (r = 0.22, p < 0.05), negatively correlated with *ERCC1* (r = − 0.25, P < 0.05). *BRAF V600E* was positively correlated with Sb (r = 0.32, p < 0.01), Tl (r = 0.27, p < 0.01), CA19-9 (r = 0.55, p < 0.001), NSE (r = 0.41, p < 0.001), AFP (r = 0.49, p < 0.001) and CK19 (r = 0.66, p < 0.001). *XRCC1 (rs25487)* was positively correlated with Se (r = 0.24, p < 0.05), NSE (r = 0.38, p < 0.01) and CK19 (r = 0.52, p < 0.001), negatively correlated with Co (r = − 0.23, P < 0.05). For heavy metals, V was positively correlated with Cr (r = 0.32, P < 0.01), Ni (r = 0.5, P < 0.001) and Hg (r = 0.33, P < 0.001); Cr was positively correlated with Cu (r = 0.21, P < 0.05) and Hg (r = 0.35, P < 0.001); As was positively correlated with Ba (r = 0.77, P < 0.001), Tl (r = 0.49, P < 0.001) and Cd (r = 0.2, P < 0.05); In addition to the above, other heavy metals also showed significant correlations, as shown in Fig. 3. Moreover, there were also significant correlations between the 5 biomarkers. Specifically, CA19-9 was positively correlated with CEA (r = 0.33, P < 0.01), while NSE was positively correlated with AFP (r = 0.82, P < 0.001) and CK19 (r = 0.89, P < 0.001). In addition, AFP displayed a positive correlation with CK19 (r = 0.93, P < 0.001).

### Distribution of XRCC1 (rs25487) polymorphism and its correlation with MSI status

The genotype and allele frequency distribution of *XRCC1 (rs25487)* polymorphism in MSI-L, MSI-H and MSS are shown in Table 5. Of the 101 CRC patients, 57 (56.44%) carried CC genotype, 39 (38.61%) carried CT genotype, and 5 (4.95%) with T/T genotype. The T and C allele frequencies of *XRCC1 (rs25487)* were 24.26% and 75.74%, respectively. Notably, there was a significant correlation between *XRCC1 (rs25487)* polymorphism and MSI status (P<0.05).

**Table 5:** Distribution of genotype and allelic frequency of polymorphisms of *XRCC1 (rs25487)* according to MSI level

SNPs	Genotype	Group			Total	*p* value
		MSI-H	MSI-L	MSS		
*XRCC1 (rs25487)*	CC	5 (31.25)	7 (50)	45 (63.38)	57 (56.44)	0.022
	CT	8 (50)	7 (50)	24 (33.80)	39 (38.61)	
	TT	3 (18.75)	0 (0)	2 (2.82)	5 (4.95)	
Allele Frequency (%)	T	43.75	25	19.72	24.26	
	C	56.25	75	80.28	75.74	

### Relationship between BRAF mutation and heavy metal level in CRC patients

**Fig. 4:**
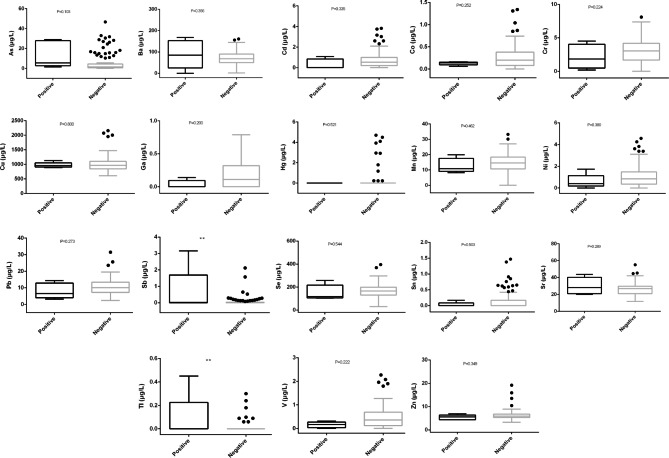
Comparison of 18 heavy metals levels between the *BRAF V600E* negative group and the *BRAF V600E* positive group. *, P < 0.05; **, P < 0.01; ***, P < 0.001; ****, P < 0.0001

Then we explored the relationship between *BRAF V600E* mutation and the heavy metal levels, and the results showed that the levels of Sb and Tl were significantly higher in the positive group compared with the negative group (p < 0.01), while the concentrations of other heavy metals (including As, Ba, Cd, Co, Cr, Cu, Ga, Hg, Mn, Ni, Pb, Se, Sn, Sr, V and Zn) were not significantly different between the two groups (p > 0.05) (Fig. 4).

## Discussion

In the present study, the levels of 18 heavy metals were compared between 101 CRC patients and 60 healthy controls, and the results showed that patients in the CRC group had the significantly higher levels of Cr, Cu and higher levels of V, As, Sn, Ba, Pb but a significantly lower level of Se compared with the control group. Spearman correlation analysis further showed that CRC risk was positively correlated with the levels of V, Cr, Cu, As, Sn, Ba and Pb, but negatively correlated with Se. Moreover, the logistic regression analysis demonstrated that Cr, Cu, As and Ba were determined as the independent risk factors of CRC after adjusting different influencing factors (including age, gender, smoking, drinking). A meta-analysis showed that the International Agency for Research had identified As and Cr as carcinogens [33]. Study found that there was a higher level of Cu but a lower level of Se in thyroid cancer patients compared with the healthy controls, consistent with our results [34]. As an essential trace mineral, Se has a wide impact on human health, including the development of cancer [35]. It has been reported that Se plays a protective role in the development of thyroid cancer, while As will destroy its anticancer activity [34, 36, 37]. A study on the correlation between Se and cancer mortality, it was found that there was a strong negative correlation between the level of Se and cancer mortality such as CRC [26]. It is also reported that supplementation with Se plays an important role in resisting viral diseases, immune function and reducing inflammation [38]. As a category I human carcinogen, the exposure of As is associated with an increased risk of cancer, including lung, skin, liver, prostate and bladder cancer [39]. Cu is not only the key component of many essential enzymes, but also the key regulator of cell signal transduction pathway, and plays a role in many biological processes [40, 41]. High levels of Cu can lead to cancer progression including cancer proliferation, angiogenesis and metastasis [42]. Many studies have confirmed that the level of Cu in both tumor and serum of cancer patients was significantly higher than that of healthy people [43]. In addition, elevated Cu levels are associated with multiple cancers, including ovarian, bladder, breast, lung, cervical, oral, pancreatic, gastric, and thyroid cancer [42]. It is worth noting that the increase of serum Cu level is related to the cancer stage and progression of breast and CRC [44]. Several studies have shown that the level of toxic metals such as Cu increases in the whole blood of patients with CRC, while the level of Se decreases, and there was a significant correlation between various metal elements, consistent with our research, indicating that these elements and their interactions may play a role in the development of CRC [45, 46]. Besides, it was demonstrated that there was a significant positive correlation between Cu and the risk of gastric cancer (GC) [47]. Other studies found that the increase of blood Pb level not only significantly increased the risk of lung cancer mortality, but also positively correlated with the risk of urologic neoplasms or digestive tract cancer [48, 49]. Li et al. reported that plasma Cu, Cr, Pb, Mn and Ni were significantly associated with incident cancer risk in T2DM patients [49]. Some studies have shown that V has genotoxicity, nephrotoxicity, hepatotoxicity, neurotoxicity, cardiotoxicity and carcinogenesis [50]. The increase of V level may be a risk factor for cancer development, and the frequency of neoplasia is positively correlated with the susceptibility to V-induced inflammation [50]. The above researches were consistent with our results.

Besides that, the characteristics of CRC patients in 30 patients with MSI and 71 patients with MSS were compared. Results showed that tumor stage, tumor size, vascular invasion, lymph node metastasis, *B-RAF V600E* and *NRAS* Codon 12/13 were significantly different between the two groups. Then Spearman correlation analysis among MSI, *BRAF V600E*, *ERCC1*, *XRCC1 (rs25487)*, 5 biomarkers, and 18 heavy metals illustrated that MSI was positively correlated with *BRAF V600E* and *XRCC1 (rs25487)*, while negatively correlated with *ERCC1*. For, *BRAF V600E*, it was positively correlated with Sb, Tl, CA19-9, NSE, AFP and CK19. *XRCC1 (rs25487)* was positively correlated with Se, NSE and CK19, negatively correlated with Co. *ERCC1* has a critical function in the nucleotide excision repair (NER) pathway and plays a vital role in DNA repair [14]. Jiang et al. proved that *ERCC1* was highly expressed in CRC patients [51]. In addition, in the study of *ERCC1* in postoperative non-small cell lung cancer, it was found that the expression of *ERCC1* mRNA was negatively correlated with chemotherapy efficacy and survival time of patients [52]. Studies have shown that CRC patients with MSI have a better prognosis compared to MSS [31, 53]. BRAF is a downstream gene of RAS in the RAS-RAF-MAPK signaling pathway, *BRAF V600E* mutation leads to uncontrolled cell proliferation, migration, escape from apoptosis and angiogenesis [54, 55]. Research showed that BRAF is associated with poor prognosis in CRC patients, especially in *BRAF V600E* MSS patients [56–58]. MSI is one of the main carcinogenic pathways of CRC [59]. MSI features and *BRAF V600E* mutations often occur simultaneously in CRC, which means that there is a strong correlation between MSI status and *BRAF V600E* mutations in CRC [60, 61]. In the present study, MSI was positively correlated with *BRAF V600E*, and the mRNA expression level of *ERCC1* was significantly higher in MSS compared to MSI. In addition, we explored the relationship between *BRAF V600E* mutation and the heavy metal levels, and the results showed that the levels of Sb and Tl were significantly higher in positive group compared with the negative group. CA19-9 and CEA are two weighty tumor markers commonly used in gastrointestinal malignant tumors, and their elevated levels are associated with CRC and advanced colorectal neoplasia [62]. CK19 is a suitable marker for detecting cancer cells and can be used as a prognostic indicator for cancer patients [25]. Recent research shows that CK19 can enhance the tumorous properties of colon cancer, breast cancer and hepatocellular carcinoma, proving that CK19 plays an important role in carcinogenesis [63]. The protein encoded by the *XRCC1* gene plays an important role in the base excision repair pathway [64]. Studies have shown that *XRCC1 (rs25487)* polymorphism is associated with an increased risk of CRC [13]. Our results showed that there was a significant correlation between *XRCC1 (rs25487)* polymorphism and MSI status. The results of Iarmarcovai et al. showed that *XRCC1* variant allele coding Gln amino acid at position 399 *(rs25487)* showed a higher number of DNA breaks in people who exposed to heavy metals such as Co [65]. However, until now, there has been very little research on the relationship between *XRCC1* and heavy metals. In our research, *XRCC1 (rs25487)* was found to be positively correlated with Se but negatively correlated with Co.

However, current research still has some limitations. Firstly, the sample size of this study is relatively small, which may limit the generalizability of the findings. Secondly, although we have adjusted for potential confounding factors to assess the risk of CRC, we cannot completely exclude the impact of unmeasured confounding factors or reverse causal relationships. Therefore, these results require further expansion of the sample size for large-scale validation.

## Conclusion

To our knowledge, this is the first study to investigate the correlation between heavy metal elements, MSI, tumor markers, and genetic polymorphism in patients with CRC. We found that Cr, Cu, As and Ba were the risk factors for CRC. Low level of Se and high levels of V, As, Sn, Ba, Pb, Cr and Cu may increase the risk of CRC. MSI was positively correlated with *BRAF V600E*, negatively correlated with *ERCC1*, *BRAF V600E* was positively correlated with Sb, Tl, CA19-9, NSE, AFP and CK19, which indicated that Sb and Tl may cause *BRAF V600E* mutations, leading to MSI. *XRCC1 (rs25487)* was found to be positively correlated with Se but negatively correlated with Co. The expression of *ERCC1* may be related to MSS, while the *XRCC1 (rs25487)* polymorphism is related to MSI. However, this observation needs to be confirmed in larger cohorts in future studies.

## Data Availability

The data sets generated and/or analysed during the current study are available from the corresponding author on reasonable request.
